# Developing patient journey maps with Aboriginal and Torres Strait Islander peoples living with dementia or cognitive impairment and their carers: protocol

**DOI:** 10.1136/bmjopen-2024-090672

**Published:** 2025-05-08

**Authors:** Penny O’Brien, Craig Sinclair, India Kinsey, Jesse Zanker, Madeleine Juhrmann, Robyn Smith, Sandra Thompson, Dawn Bessarab, Dina Lo Giudice

**Affiliations:** 1 Department of Medicine, Royal Melbourne Hospital, The University of Melbourne, Melbourne, Victoria, Australia; 2 School of Psychology, University of New South Wales, Randwick, New South Wales, Australia; 3 Neuroscience Research Australia (NeuRA), Sydney, New South Wales, Australia; 4 University of New South Wales, Sydney, New South Wales, Australia; 5 Research Centre for Palliative Care, Death and Dying (RePaDD), College of Nursing and Health Sciences, Flinders University, Adelaide, South Australia, Australia; 6 Improving Palliative, Aged and Chronic Care through Clinical Research and Translation (IMPACCT), University of Technology Sydney, Sydney, New South Wales, Australia; 7 Combined Universities Centre for Rural Health, The University of Western Australia, Perth, Western Australia, Australia; 8 Centre for Aboriginal Medical and Dental Health, University of Western Australia, Perth, Western Australia, Australia

**Keywords:** Dementia, Life Change Events, Cognition

## Abstract

**Abstract:**

**Introduction:**

Although Aboriginal and Torres Strait Islander peoples are increasingly living healthier and longer lives, they continue to experience a high prevalence and incidence of dementia and cognitive impairment. Navigating dementia care services is challenging, and there is limited availability of flexible, culturally secure health and community care services. The aim of this study is to use a culturally adapted patient journey mapping methodology to examine the lived experiences of Aboriginal and Torres Strait Islander Aboriginal peoples living with dementia/cognitive impairment and their carers navigating their care journeys.

**Methods and analysis:**

The overarching principle guiding this project is cultural security, referring to the incorporation of processes such that the research will not compromise the cultural rights, values and expectations of Aboriginal and Torres Strait Islander peoples. In this three-phase participatory action research study, we will (1) formalise relationships with health and home care services as recruitment sites; (2) conduct research yarns (a culturally secure qualitative data collection tool) with Aboriginal and Torres Strait Islander peoples living with dementia or cognitive impairment and their carers about their experiences of healthcare including perceived barriers and enablers to high-quality care. Data collected in research yarns will be analysed using a modified framework approach to map patient journeys and; (3) make recommendations for improving care identified by participants to be discussed and refined with stakeholder groups and to inform best practice guideline development.

**Ethics and dissemination:**

This project follows the National Health and Medical Research Council’s guidelines for ethical conduct in research with Aboriginal and Torres Strait Islander communities and has been designed with active involvement and governance by Aboriginal and Torres Strait Islander peoples. The results will be disseminated through community feedback sessions, newsletters, conference presentations, peer-reviewed publications and best practice guidelines. Dissemination will also be guided by an established Aboriginal Reference Group.

**STRENGTHS AND LIMITATIONS OF THIS STUDY**

- This project involves multilevel engagement of Aboriginal and Torres Strait Islander peoples and participatory action research approaches to codesign strategies to improve dementia healthcare delivery.
- This project is governed by an Aboriginal and Torres Strait Islander Community Reference Group, who have provided input into the study design and methodology.
- This project uses a novel ‘River of Life’ patient journey mapping methodology that has been culturally adapted for use with Aboriginal and Torres Strait Islander people, an approach that has not been used previously in this context.
- Geographical coverage and participant representation may be difficult to achieve due to potential travel requirements and the time-consuming nature of the research.

## Introduction

 Dementia describes a collection of conditions characterised by cognitive impairment and changes in brain function that impact thinking, memory and ability to perform activities of daily living.^1^ Population ageing and changing social determinants of health are anticipated to manifest in a greater future burden of dementia, such that the number of people living with dementia, disability, carer burdens and healthcare costs are projected to grow exponentially over the next 30 years.^2^ The Aboriginal and Torres Strait Islander population (respectfully Aboriginal peoples herewith, see box 1) is growing, with projections indicating a near doubling of Aboriginal peoples over the age of 55 years in the period between 2011 and 2026.^3^ Many older Aboriginal peoples play an important role in the health and well-being of their communities. This includes roles as Elders (an Aboriginal and/or Torres Strait Islander person who is highly respected and recognised in their community as a custodian of cultural knowledge) as well as family caregiving roles and providing leadership to their wider communities.^4^ Although Aboriginal peoples are increasingly living to older ages, they continue to experience a high prevalence of dementia, with rates of up to five times than those documented among non-Indigenous Australians.^4 5^ Aboriginal peoples are also more likely to develop dementia at a younger age.^3^ High prevalence rates of dementia, young-onset dementia and cognitive impairment may be due, in part, to potentially modifiable risk factors and comorbid conditions, such as hypertension, diabetes mellitus, obesity, smoking and head injury.^1^ Multimorbidity also contributes to poorer quality of life^6^ and difficulty accessing health services.^7^

Box 1 **A note on language**

Terminology regarding Aboriginal and Torres Strait Islander identity is varied and complex. The Indigenous peoples of Australia are Aboriginal and Torres Strait Islander peoples, a diverse population representing over 250 language groups. In this paper, we refer to Aboriginal and/or Torres Strait Islander peoples as Aboriginal peoples. We acknowledge the diversity of cultures of all Aboriginal and Torres Strait Islander peoples in Australia.

Aboriginal peoples continue to experience complex health needs and barriers to appropriate healthcare due to the enduring legacy of colonisation and associated race-based government policies in Australia.^8^ The complex health and social needs of Aboriginal peoples are well known and well documented; improving access to high-quality healthcare that meets the needs, values and preferences of Aboriginal peoples is paramount to improving well-being. The 2014–2015 National Aboriginal and Torres Strait Islander Social Survey found that 22% of Aboriginal peoples over the age of 15 years living in non-remote areas experienced difficulty accessing healthcare services, which was almost 10 times higher than the wider population.^9 10^ Delivering high-quality dementia care, including support for carers, is also complex. Several types of service provisions, community services and supports are often required as dementia progresses, and often people living with dementia experience a broad range of care transitions from diagnosis, home, hospital, residential care and palliative care settings.^11^,^13^ For Aboriginal peoples, navigating dementia care services is made challenging by the increased burden of multimorbidity, location of services, issues related to health provider communication, cooperation among services and between individual services and the community, the need for community, carer and staff education and training and the limited availability of flexible, culturally secure health and community care services.^14 15^ In addition to complex healthcare journeys, the necessity of culturally responsive services to support Aboriginal peoples living with dementia at the end of their life, including palliative care, end of life care and advance care planning (ACP), is a priority that has been highlighted in the Aboriginal and Torres Strait Islander Dementia Research and Translation Roadmap, National Palliative Care Strategy and identified by Elders as an important aspect of improving well-being.^16 17^ ACP is a way of identifying and sharing life values and goals to ensure medical and lifestyle decisions align with a person’s wishes in the event that they are unable to make those decisions in the future. ACP discussions and associated documents need to be communicated and recorded in culturally meaningful ways.^18^

A better understanding of the lived experience of Aboriginal peoples living with dementia and cognitive impairment and their carers is needed to inform and enhance service provision and improve clinical outcomes and well-being. Patient journey mapping is a novel, evolving approach used to gain insight into how individuals experience and navigate complex and dynamic health systems and highlight gaps in access, continuity and quality of healthcare.^19^,^22^ In this context, we define ‘navigate’ as searching for, identifying and coordinating any health or social care services.^23^ Davies *et al* define the patient journey mapping process as a “patient-oriented project that has been undertaken to better understand barriers, facilitators, experiences, interactions with services and/or outcomes for individuals and/or their carers and family members as they enter, navigate, experience and exit one or more services in a health system by documenting elements of the journey to produce a visual or descriptive map” (Davies *et al*, p84).^21^ This protocol outlines the methodology for a study to examine the lived experiences of Aboriginal peoples living with dementia/cognitive impairment and their carers navigating their care journeys.

## Project context and setting

### The Teaching, Research, and Community Knowledges (OnTRACK) programme

This protocol outlines research that will be undertaken within the OnTRACK programme, a National Health and Medical Research Council (NHMRC) Centre for Research Excellence established to promote brain health among Aboriginal peoples. The OnTRACK team is a national interprofessional group of Aboriginal and Torres Strait Islander and non-Aboriginal academics, clinicians and health service staff working collaboratively to optimise the health and well-being of older Aboriginal peoples at risk of or living with dementia, their carers, families and communities. Our authorship team includes two Aboriginal researchers (DB, a Bard/Yjindjarbandi woman and research leader; IK, a Warrawong doctor).

### Governance: OnTRACK Aboriginal and Torres Strait Islander Community Reference Group (CRG)

This project will be governed by the OnTRACK Project Management group and OnTRACK Aboriginal and Torres Strait Islander CRG, which have already been established through previous projects conducted by members of the research team. This group includes Elders, older Aboriginal peoples, carers of Aboriginal peoples living with dementia, Aboriginal health or care workers and advocates with relevant expertise. The CRG provides input and oversees the project activities including advice on the strategic direction, cultural security, research methods, interpretation and dissemination of the research findings. Cultural security is both a methodological and ethical consideration that emphasises that Aboriginal health research must be conducted in a manner that prioritises Aboriginal and Torres Strait Islander cultural values, beliefs and ways of knowing and doing, and therefore will not compromise the cultural rights, expectations, needs and preferences of Aboriginal peoples.^24^ The CRG will meet three times a year, either face to face or through a videoconferencing platform as needed.

## Methods and analysis

### Overview

**Figure 1:**
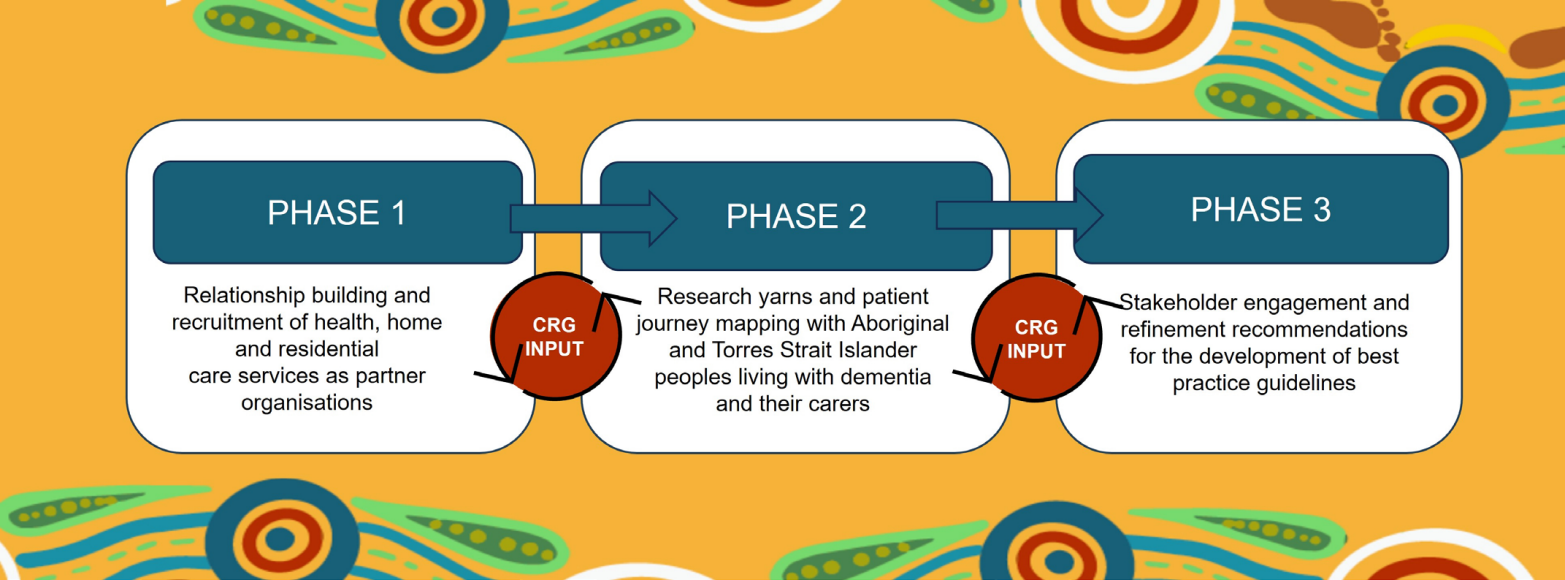
Research process overview. CRG, community reference group.

This patient journey mapping project will be conducted in three phases (see figure 1) between February 2025 and April 2027. It will be reported in accordance with the Consolidated Criteria for Reporting Qualitative Research checklist^25^ and the Aboriginal and Torres Strait Islander Quality Appraisal Tool.^26^ In phase I, we will formalise relationships with health and home care services and residential aged care services, with a focus on Aboriginal Community Controlled Health services (ACCHs) as recruitment sites. In phase II, research yarns^27^ (a culturally secure qualitative data collection methodology) will be conducted with Aboriginal peoples living with dementia or cognitive impairment and their carers/key family members about their experiences of healthcare including perceived barriers and enablers to high-quality, culturally secure care. Data collected in research yarns will draw on ‘River of Life’ methodology^28^ and will be analysed using a modified framework analysis approach^29 30^ to map patient journeys. This will enable the identification of gaps in healthcare provision, barriers and enablers at points along the healthcare journey to culturally secure dementia care and an exploration of the impact of these journeys on the health and well-being of Aboriginal peoples living with dementia or cognitive impairment and their carers, family and communities. In phase III, recommendations for improving care identified by participants and through the data analysis process in phase II will be presented to stakeholder groups, including Aboriginal peoples living with cognitive impairment and dementia, Aboriginal health providers and clinicians who deliver care to older Aboriginal peoples as well as the OnTRACK CRG for further exploration. Recommendations will be discussed and refined with the ultimate aim of informing the development of best practice guidelines.

### Aims and objectives

The aims are to use a culturally adapted patient journey mapping process (River of Life) to explore and understand the lived experiences of Aboriginal peoples living with dementia or cognitive impairment and their carers navigating their care journeys, including barriers and enablers to receiving high-quality care. The objectives are:

1. To explore and understand patient journeys for Aboriginal peoples living with dementia or cognitive impairment and multimorbidity.
2. To explore barriers and enablers to high-quality healthcare experienced by Aboriginal peoples living with dementia or cognitive impairment from the perspective of patients, their carers, families and local healthcare providers.
3. To explore and understand communication processes, support and decision-making in relation to ACP and end of life care for Aboriginal peoples living with dementia or cognitive impairment.
4. To identify complexities, critical steps and gaps in healthcare delivery for Aboriginal peoples living with dementia or cognitive impairment to inform the codesign and development of recommendations to improve healthcare delivery for Aboriginal and Torres Strait Islander people in relation to multimorbidity, end of life care and ACP.

### Study design

#### Participatory action research (PAR) framework

This project will be guided by a culturally adapted PAR framework, which is used for conducting research that prioritises researchers and participants generating and sharing knowledge collaboratively while working towards pragmatic and positive outcomes.^31^ Although many forms of PAR exist, common components of this methodology include being cyclical in nature and involving phases of both research and action.^32 33^ One example, described by Stringer, revolves around the three steps: look (data gathering), think (analysis and interpretation) and act (resolving problems—planning and implementing sustainable solutions).^32^ This PAR framework has since been culturally adapted in collaboration with Aboriginal women and health professionals.^33^ Although first coproduced with Aboriginal women within a study specific to women’s health, this culturally adapted framework has since been used effectively to guide PAR involving both Aboriginal and Torres Strait Islander men and women.^33^ The first step, ‘look’, was reconceptualised as ‘look and listen’, to emphasise listening and hearing Aboriginal and Torres Strait Islander community members and health professionals. The second step was renamed ‘think and discuss’ to emphasise the importance of collective decision-making. The third phase, ‘act’ became ‘take action’, to provide an energetic and pragmatic focus.^33^ A culturally adapted, collaborative PAR approach with repeated cycles of look and listen, think and discuss and take action will guide the OnTRACK patient journey mapping project.

### Participants and recruitment

#### Health, home and residential care services

The OnTRACK programme has existing, established partnerships with primary care services, hospitals and ACCHs in metropolitan, regional and remote locations across Australia, as well as Aboriginal residential aged care services such as the Aboriginal Community Elders Services. Drawing on these existing partnerships, we will formally invite health services (including health services, ACCHs and residential aged care services) to participate in the OnTRACK patient journey mapping project. An email containing project information, a formal letter of invitation and examples of participant information and consent forms will be sent to each health service. A member of the OnTRACK research team will then follow-up with and meet a representative of each health service to discuss the research in more detail. If the service agrees to participate as a partner site, a Memorandum of Understanding document will be developed and agreed on by each of the participating health services, the OnTRACK CRG and OnTRACK project management team. We will aim to recruit a total of 5–6 diverse health services as partner sites across a range of geographical settings (metropolitan, regional and remote), and organisational consent will be sought by health services managers.

#### Aboriginal peoples living with dementia or cognitive impairment and their carers

This participant group will include Aboriginal peoples living with dementia or cognitive impairment and their carers, key family members or a support person (table 1). Health service staff members of partner health services will be asked to identify potential participants from medical records. Health service staff or clinicians known to potential participants will be the first to make contact and describe the study, either face to face at the health service or via phone. Potential participants and/or their carers/family members will be asked by the health service staff member if they agree to an Aboriginal and/or Torres Strait Islander member of the research team contacting them about the project. If they agree, an Aboriginal and/or Torres Strait Islander member of the research team will contact potential participants to yarn in more detail about the patient journey mapping project and invite them to participate. Aboriginal peoples living with dementia or cognitive impairment and their carers/key family members will be provided with a participant information sheet that balances the need for detailed information about the project, readability and visual clarity.^34^ We will also offer flexibility in formats to receive study materials (written, oral) and where appropriate use local Aboriginal language interpreters and translation services to ensure equitable opportunities to participate.

**Table 1:** Inclusion and exclusion criteria for Aboriginal peoples living with dementia or cognitive impairment

Inclusion criteria	Exclusion criteria
Identify as Aboriginal and/or Torres Strait Islander	Do not identify as Aboriginal and/or Torres Strait Islander
Diagnosis of dementia or cognitive impairment	Not able to give consent or are non-consenting
Have a primary carer, family member or key support person involved in their care who agrees to participate in the research	Experiencing responsive behaviours or psychiatric symptoms that may increase risk of distress for the participant or place researchers at risk
Have been a primary carer of an Aboriginal and Torres Strait Islander person who had a diagnosis of cognitive impairment or dementia who has since died	Current or recent mental health crisis, including bereavement or major trauma, especially where involved risk of self-harm or harm to others
Be able to give verbal or written informed consent as deemed by the local treating team	Acutely unwell
Adults 18+ years of age	

Participants in this group will have a diagnosis of cognitive impairment or dementia (as diagnosed by their treating clinician or/and medical records), therefore advice from the local treating team/a trained clinician will be sought to determine if potential participants are able to be approached (ie, permission to access the person with dementia) by the research team. Permission to access is one of the first elements of ‘process consent’ as described by Dewing.^35^ The method of process consent comprises five non-linear elements including background and preparation, establishing the basis for capacity, initial consent, ongoing consent monitoring and feedback and support.^36^ Process consent is said to enable greater and more meaningful involvement of people with cognitive impairment in research by promoting more active participation in the consent process and further enhancing aspects of reciprocity and continuity in regard to the research relationship.^36^ If the person with cognitive impairment or dementia is not able to give consent, only their carer/family member will be approached to participate. We will aim to recruit a total of 20 participants (including individual participants or participant–carer dyads or carers) from a range of geographical locations (rural, remote, metropolitan), who are at different points of their healthcare journey (eg, 6–7 who have been recently diagnosed, up to 1 year post diagnosis, 6–7 participants 1–3 years post diagnosis and 6–7 people 3+ years from diagnosis). Through our yarning approach, we anticipate that each interview will generate rich data. Although we have stated 20 as an approximate sample size, we will be guided by thematic saturation within each group, that is, the point at which no new themes are emerging from the data.^37^ All are likely to have a range of comorbid conditions such as cardiac conditions, diabetes, kidney disease and chronic obstructive pulmonary disease.

### Data collection

#### Research yarning and River of Life methodology

Aboriginal and Torres Strait Island peoples living with cognitive impairment or dementia and their carers/key family members will participate in research yarns about their experiences of healthcare including barriers and enablers to quality care. Research yarning is acknowledged as a culturally secure approach to collect qualitative data as it aligns with Aboriginal ways of knowing and doing, such as the use of storytelling.^27^ Researchers conducting research yarns must prioritise the lived experience and cultural values of Aboriginal peoples by building relationships with Aboriginal and Torres Strait Islander participants and ensuring yarns are informal and conversational. Research yarns will be conducted by an Aboriginal and/or Torres Strait Islander graduate research student or researcher face to face at a location convenient to participants (in their home, their local ACCH) or via phone or teleconferencing. In the case of participant–carer dyads, yarns will be conducted together. Participants will also be able to request additional support people or family members to be present during research yarns. Members of the research team will travel to the location of the participants where feasible. Prior to the yarn, participants will be provided with an information sheet that will describe the process of the research yarn, including the ‘River of Life’ approach and example yarning guide questions.

All research yarns will begin with a social yarn to build trust and rapport between the researchers and the participants. Following the social yarn, the research topic yarn will begin. The research topic yarn will draw on the ‘River of Life’ method,^28^ a PAR approach that uses metaphors to assist participants to reflect on past experiences. The River of Life methodology was originally designed as a reflective tool which uses the metaphor of a river to assist people to reflect on their life story. The methodology will be adapted to assist patients to reflect on their care experiences. In collaboration with the OnTRACK CRG, we have adapted this methodology to be used in an Aboriginal health context. Aboriginal peoples are a diverse population representing over 250 language groups.^38^ Depending on each Aboriginal and Torres Strait Islander person’s cultural identity, they may associate with being Freshwater Peoples, Saltwater Peoples, Desert Peoples or Mountain Peoples. Therefore, participants will be able to choose a natural landscape (river, ocean, walking on country or mountains/valleys) that resonates with them as a metaphor for their journey.

Different aspects of each natural landscape will be used to highlight critical moments in participants’ healthcare journeys. Using a river as an example, a boulder may represent a barrier or a challenge; rapids may represent a fast-moving or well-facilitated part of their journey and estuaries or connected streams may represent relationships or additional health services they encountered on their journey. Metaphors and language used to describe the river to participants will be informed by the OnTRACK CRG to ensure language is culturally appropriate. During the research topic yarn, participants will be asked to describe their patient care journeys, including their symptoms and experiences that led to their initial diagnosis of cognitive impairment and/or dementia, barriers and enablers of seeking care, their healthcare experiences including managing chronic and comorbid conditions and their experiences and perception of ACP and end of life care. Research topic yarns will be guided by a semistructured yarning schedule and cover participants’ experiences prior to diagnosis, current experiences and future planning, as well as their ideas for healthcare improvements to challenges they may have experienced. Yarning schedules will allow for flexibility for participants to raise additional relevant information, or for researchers to explore additional lines of inquiry. Online supplemental appendix 1 outlines an example interview schedule. Research yarns will be audio recorded with participants’ consent, and those who do not wish to be audio recorded will not be excluded from participating. If participants do not consent to audio recording, researchers will take detailed notes during the yarn. Participants who participate in research yarns will each be reimbursed for their time with a $AUD100 gift card.

### Data analysis plan

Audio data collected during research yarns will be transcribed by the research team, a trusted Australian external transcription service or by using a secure online artificial intelligence speech to text application (Sonix.Ai). A modified framework approach,^29^ summarised in table 2, will be used to identify themes relating to conceptual frameworks for older Aboriginal peoples’ well-being and access to care such as the Good Spirit Good Life tool^39^ and Davy’s framework for access to primary care for Aboriginal peoples.^40^ We will also map patient journeys chronologically using a patient journey mapping tool adapted from the Lowitja Institute’s Managing Two Worlds Together project.^41^ This information will be interpreted and mapped on to a visual representation of their journey (such as a river) with the support of an online whiteboarding application (Miro). By using comprehensive conceptual frameworks of well-being and access to care, we will highlight aspects of people’s well-being affected by living with dementia and identify gaps and opportunities for improvements in healthcare delivery. Researchers will follow-up with all participants to conduct member checking, to ensure that the researchers’ interpretations of patient journeys accurately represent participant experiences.^42^ Members of the research team will collaborate with the OnTRACK CRG before and during data analysis and make adjustments to the methods based on the OnTRACK CRG’s recommendations.

**Table 2:** Framework analysis plan

Data analysis step		Description
Step 1	Familiarisation	The primary researcher becomes familiar with transcripts by reading and re-reading the transcripts.
Step 2	Open coding	Two researchers (at least one Aboriginal and/or Torres Strait Islander researcher) code the transcripts inductively using open coding, identifying broad concepts relating to the healthcare experiences of Aboriginal peoples living with cognitive impairment or dementia.
Step 3	Adapting/developing thematic framework	Salient themes identified through open coding will be refined into a thematic framework which also encapsulates the domains of the Good Spirit Good Life tool and Davy’s access to care framework.
Step 4	Charting	The thematic framework will be applied to each transcript, which will be summarised using a matrix in Microsoft Excel. The matrix will consist of codes including predetermined well-being/access domains in the left-hand column and chronological patient journey moments along the top row. Important moments from within each transcript will be extracted into each timepoint/code and inserted into the corresponding cell in the matrix. This step will allow the research team to understand the relationship of codes between and within each patient journey and note similarities and differences between them.
Step 5	River of life	Each matrix will be summarised using a visual depiction of the journey. Main themes will be plotted along the visual representation of the journey.
Step 6	Member checking	Researchers will follow-up with each participant/carer dyad to check that the summarised, visual representation of their patient journey accurately represents their patient journey.
Step 7	Composite patient journey map	Drawing on composite storytelling,^30^ we will use data from each patient journey map to construct a single journey representing an amalgam of all participants included in the study. This map will be developed to capture an authentic and anonymous account of the complex, multidimensional and interconnected nature of lived experience of health systems by highlighting salient themes identified in the dataset.

#### Stakeholder engagement

Recommendations for improving care will be based on addressing issues identified by participants, and via data analysis, will be presented to stakeholder groups, including Aboriginal peoples living with cognitive impairment and dementia, Aboriginal health providers and clinicians who deliver care to older Aboriginal peoples as well as the OnTRACK CRG for further exploration.

### Public and patient involvement

This project is predicated on meaningful patient and public involvement. An established Indigenous Reference Group, comprised of Elders, older Aboriginal peoples living with dementia and their carers, Aboriginal health or care workers and advocates with relevant expertise, will guide the conduct of all parts of this research, including the processes reported in this protocol.

## Ethics and dissemination

### Ethical considerations

This project follows the NHMRC’s guidelines for ethical conduct in research with Aboriginal peoples and communities for researchers and stakeholders^43^ and has been designed with Aboriginal and Torres Strait Islander involvement and governance structures across multiple levels. Each of the six core values of Spirit and Integrity, Cultural Continuity, Equity, Reciprocity, Respect and Responsibility has been addressed in a University of Melbourne ethics application (under review, ID 25287); additional state-based ethics approvals will be obtained where required. The overarching principle guiding this project is cultural security.^24^ We will ensure cultural security through multilevel and ongoing Aboriginal and Torres Strait Islander governance and involvement throughout all phases of the project to guide all methodological and cultural aspects of the research. This will also ensure the research responds to community identified priorities and builds the capabilities of Aboriginal and Torres Strait Islander researchers to work in brain health and non-Aboriginal research and clinicians to work within Indigenous research paradigms. We will also adopt culturally secure research methods such as the use of PAR processes and research yarning.^27^

### Dissemination

Findings from this study will inform the development of comprehensive best practice guidelines on culturally responsive dementia care, including challenges relating to multimorbidity and approaches to ACP and end of life care. We will develop resources using a codesign approach for community awareness raising and capacity building within the Aboriginal health workforce, including aged and dementia care and palliative care. The wider OnTRACK programme has a strong translation focus that will ensure wide dissemination of research findings and clinical guidelines in key policy arenas, continuing our effective, established approaches such as briefing documents to government, joining government advisory groups, actively participating in consultations and roundtables focusing on dementia nationally and internationally, and working with peak bodies to maximise promotion and translation of research findings.

Further dissemination strategies will be guided by the OnTRACK CRG and are likely to include regular feedback to individual research participants and health services, peak bodies and stakeholder groups, government and general public via media as well as community feedback sessions, newsletters, academic publications and national and international conferences/forums. All partner sites/organisations will be encouraged to participate with the project team in the process of developing reports and dissemination approaches that are relevant to their own community and networks.

## Supplementary material

10.1136/bmjopen-2024-090672 online supplemental file 1
