# Supplementary material for: Developing patient journey maps with Aboriginal and Torres Strait Islander peoples living with dementia or cognitive impairment and their carers: protocol
Source: BMJ Open. 2025 May 8;15(5):e090672. doi: 10.1136/bmjopen-2024-090672 (PMC12067768; doi:10.1136/bmjopen-2024-090672)
Supplement: online supplemental file 1 [file bmjopen-15-5-s001.docx]

**Supplemental Material**

**Appendix 1**

| ***Aim: To explore and understand the lived experiences of Aboriginal peoples living with dementia or cognitive impairment and their carers navigating their care journeys, including barriers and enablers of receiving high-quality healthcare.*** | |
| --- | --- |
| **SOCIAL YARN** | |
| **RESEARCH YARNING QUESTIONS** | |
| **Introduction and socio-demographics** | - Acknowledgement of Country - Check received written consent and/or obtain verbal consent - Let participants know you have started the voice recorder/zoom recording - What interested you in participating? - Tell me a bit about yourself? Age, Country/language group, place of residence, occupation, hobbies? - Can you tell me a bit about your family? - What is a favourite place you have lived, worked or travelled to? |
| **RIVER OF LIFE/MAPPING ACTIVITY** | |
| **Activity description** | As part of our yarn, we will be drawing a [river/sea/Country/mountain range]* together. The landscape that you choose (e.g. river) will reflect and represent your health care journey whilst living with cognitive impairment/dementia or caring for person living with cognitive impairment/dementia.  There is no right or wrong way to do this activity – each [River of Life]* drawing will be different.  The [River of Life] drawing will be created as part of the research yarn. It can be a helpful way to recall all the things that happened along your dementia healthcare journey. The researcher will use an online program to create your River of Life drawing, and may make adjustments after the yarn. You will receive a copy of the drawing to make sure that it accurately reflects your experiences. You will be able to make changes after you received the drawing.  Let’s start yarning about your dementia health care journey. |
| **Diagnosis/symptoms** | - Can you tell me a bit about your overall health and wellbeing? - Tell me about the signs and symptoms that first appeared? - When did they start to appear?   - How many years ago?   - How old were you? What age were you? - Who noticed these signs and symptoms? - What actions or steps were taken at this time? - What health professionals/other professionals did you see when you were seeking help for the signs and symptoms you were experiencing?   - Who did you see?   - Tell me about the care that you received   - Were there any problems finding/receiving care? - Tell me about any information you received at this time? Was it helpful to you and your family? - Who else was involved in supporting you during this time? - How did you feel? |
| **Mapping 1: Diagnosis** | - How would you describe your dementia diagnosis if it was a river? - What does the start of the river look like?/What does the river look like? - Who is at the start of the river? - Does the river flow fast or slow? |
| **Health care/health professionals/supports** | - Since your diagnosis, what does your healthcare look like?   - What works well?   - What doesn’t work well?   - What has changed/is different since your diagnosis? - Can you tell me about any other supports/services you access?   - What works well?   - What doesn’t work well? - Tell me about the support around you? Who is involved in supporting you?   - Family, friends, colleagues, other people/organisations? - What is missing from your care that you’d like to have access to? - If you could change your care in any way, how would you change it? |
| **Multimorbidity** | - Do you experience any other health conditions? - How does experiencing this/these condition(s) impact you? - How does experiencing this/these conditions impact your healthcare experiences?   - Impact on dementia care   - Navigating care for multiple conditions - What works well?/doesn’t work well |
| **Mapping 2: What happens along the river?** | - What does it look like along the river? - When are there changes in the river?   - Changes in situations   - Changes in perspective   - Changes in supports/care   - Positives/challenges - Does the river have any sudden changes or bends? - Is the river long and winding? - Does the river have any boulders, rapids or waterfalls? - What does the river look like now, compared to the start? |
| *Remind participants that the next section is a sensitive topic and is about end of life care and advance care planning. Let participants know they don’t need to answer any questions if they don’t feel like it and can take breaks whenever they would like* | |
| **End of life care/advance care planning** | - Can you tell me about any yarns you have had about end of life care? - What types of supports would you like to be available at the end of your life? - Can you tell me anything you know about advance care planning/advance directives? - Can you tell me about any yarns you have had about advance care planning? - These are sensitive topics – for some people yarning about death or dying is taboo. Can you tell me any ways in which yarning about these topics could be easier for you?   - Who would you feel comfortable yarning about end of life care/advance care planning - In the event that you were not able to make healthcare decisions, how would you like your wishes to be:   - Recorded?   - Communicated?   - How could health care wishes be recorded/communicated in the most culturally meaningful ways? |
| **Mapping 3:** | - What would you like the end of the river look like? - Who would be there? - How would your values be incorporated into your care when you are |
| *NB. Researchers will discuss with participants which landscape that best reflects their journey before the research yarn and yarning guides will be updated to reflect this at the time of the yarn. | |
