# Supplementary material for: Developing patient journey maps with Aboriginal and Torres Strait Islander peoples living with dementia or cognitive impairment and their carers: protocol
Source: BMJ Open. 2025 May 8;15(5):e090672. doi: 10.1136/bmjopen-2024-090672 (PMC12067768; doi:10.1136/bmjopen-2024-090672)
Supplement: Supplementary file 2 [file bmjopen-2024-090672.reviewer_comments.pdf]

PEER REVIEW HISTORY

BMJ Open publishes all reviews undertaken for accepted manuscripts. Reviewers are asked to complete a checklist review form and are provided with free text boxes to elaborate on their assessment. These free text comments are reproduced below.

ARTICLE DETAILS

Title (Provisional)

Protocol for developing patient journey maps with Aboriginal and Torres Strait Islander peoples living with dementia or cognitive impairment and their carers

Authors

O'Brien, Penny; Sinclair, Craig; Kinsey, India; Zanker, Jesse; Juhrmann, Madeleine; Smith, Robyn; Thompson, Sandra; Bessarab, Dawn; Lo Giudice, Dina

---

VERSION 1 - REVIEW

**Reviewer**	**1**
**Name**	**Gibson, Rosemary**
**Affiliation**	**Massey University College of Humanities and Social Sciences, School of Psychology**
**Date**	**17-Sep-2024**
**COI**	**None**

Thank you for the opportunity to review this paper, Protocol for developing patient journey maps with First Nations peoples living with dementia or cognitive impairment and their carers, for consideration in BMJ Open.

This is a really important topic given the rising life expectancy among First Nation people and therefore the incidence of dementia among these populations is also rising. I believe, with minor considerations and edits (if deemed appropriate), this is acceptable for publication.

Abstract – moves from “First Nations” to “Aboriginal” people and then also “Aboriginal and Torres Strait Islander people” and then “Indigenous” people. Although this is nicely defined in box 1, it might be useful to clarify or be consistent in the abstract which needs to stand alone.

The introduction and rationale is very clear

Page 4 – lines 1-8 – do colonisation and structural racism also create barriers for First Nations people engaging or receiving healthcare..? might be useful to make clear here and cite as well – and this also feeds into the importance of your thoughtful study design including ethical and cultural considerations.

Methods – also clearly defined steps and processes including ground work involved in developing and designing the study

Needing to have a clinical diagnosis may be challenging with many of the general population underrepresented and Indigenous populations in particular whose frameworks for health, wellbeing and ageing may differ from Western medicalised models (see examples from NZ). Although it appears strong networks are already formed – perhaps some indicators of approximate numbers of First Nations people with dementia in these (or such) clinicals could be useful – to give an idea of the pool of potential participants..?

Page 11 – line 35 – can they still take part if they want to participate but not have audio recording..? – e.g. could the researchers note-take and still include their yarns?

Perhaps there is space to consider identifying any potential limitations and how the study has been designed to mitigate these

Overall, an exciting project and well written comprehensive protocol. If space (and appropriate) could be nice to include an example of the kind visual output described on page 10

I look forward to seeing the outcomes of this study

**Reviewer**	**2**
**Name**	**Kelly, J**
**Affiliation**	**University of Adelaide, School of Nursing**
**Date**	**04-Feb-2025**
**COI**	**None**

Study Protocol

Thank you for the opportunity to review this very well written and deeply considered study protocol. As per the journal, my comments and suggested revisions seek clarification or more details.

The methodology, reason for using cultural security, focusing on lived-experience and care giver experiences, using a 3 step patient journey mapping process, with multi- level engagement is well described. This strength-based approach builds on and extends existing collaborative and PAR research in an important area of care. This research builds on and has real potential to significantly contribute to First Nations, PAR, PJM and dementia research. **Abstract**

- cultural security approach and definition included
- Clear 3 step process

Terminology – well described for an international audience

I note the terminology box 'A note on language' that describes the different terms:

Good inclusion of explanations /definition of terms including Elder

I note that the Reference group is called the Indigenous Reference group, and the reason for this is provided on page 6. **Introduction**

The background and need for this study are clearly identified.

Question:

Page 4 line 42 – 44 – these modifiable risk factors and comorbid conditions are also closely linked to the ongoing impacts of colonisation, racism and discrimination/inequities. A sentence could be added referring to this, to enable the authors/project to actively counter the blaming of First Nations people for the position they find themselves in. I note you have discussed issues of access to care.

Methods

The PAR and patient journey mapping approach processes are well described. Utilising the River of Life brings a fresh and culturally responsive approach to mapping journeys. Figure 1 shows the process clearly and the inclusion of the Indigenous Reference Group throughout.

Question:

Page 7 Figure 1. Will/have the IRG also have/had input into the design of the research, & or PJM process/ tools as per statement bottom of page 12.? Perhaps another circle of IRG input could be placed before Phase 1 and after Phase 3 – this could be with a dashed circle to show overall governance of the project and processes and well as intensive involvement in the PJM specifically?

Existing partnerships and relational networks are clearly described and will help make this research feasible. Adaptations for different cultural/ family groups and English reading/ oral communication is included. The process consent has been seriously considered and is more suitable with this participant group. The numbers of anticipated participants, adapted approach and inclusion and exclusion criteria are feasible and realistic.

Research yarning and River of Life methodology

Well described extension of the research yarn that is very responsive to each person/ family/community. I can't wait to read the articles resulting from this work!

Data analysis – well thought through approach with member checking. Table 2 is effective in describing inductive and deductive analysis steps.

Ethics – also includes First Nations specific and cultural security considerations.

Dissemination, guidelines and policy development and ongoing work with peak bodies and roundtables nationally and internationally, as well as a range of knowledge translation activities including community feedback sessions, conferences and peer reviewed publications, reports and in service dissemination.

As is correct with a protocol paper – there is no results or conclusion section.

Question:

Date range – I may have missed this, but I cannot locate the dates that this project will be undertaken.

Question:

Cultural background/ experience/ roles of research team and authors. These details may have been included in other submitted documentation, and/but an included positioning of all authors, including cultural background will strengthen the paper and inform readers as to the level of First Nations researcher/community member/ lived experience and involvement.

**Reviewer**	**3**
**Name**	**Meyer, Claudia**
**Affiliation**	**Bolton Clarke Research Institute**
**Date**	**09-Feb-2025**
**COI**	**None**

Thank you for the opportunity to review this well-written protocol paper - it clearly articulates the project in a culturally secure manner and provides excellent detail of the nuances of working with First Nations people. A couple of minor points to address:

Pg 9, line 26 - how will diagnosis be confirmed?

Pg 13, lines 38-40, the emphasis on resources is on ACP/EOL - the aims of the project are to inform high-quality care overall, so is there any consideration of dissemination for this? Perhaps it is embedded within the remaining section of dissemination, but the opening sentence focuses only on ACP/EOL

There are a few small grammatical corrections to be made - pg 3 line 41, pg 4 line 14 and 36, line 53 in box 1.

VERSION 1 - AUTHOR RESPONSE

Reviewer 1

Abstract – moves from “First Nations” to “Aboriginal” people and then also “Aboriginal and Torres Strait Islander people” and then “Indigenous” people. Although this is nicely defined in box 1, it might be useful to clarify or be consistent in the abstract which needs to stand alone.

Thank you for the opportunity to clarify.

We have updated the Abstract to be consistent in our language, using the terminology Aboriginal and Torres Strait Islander peoples. We also made the decision to change our language from ‘First Nations’ to ‘Aboriginal peoples’ throughout the paper, based on feedback from the OnTRACK Aboriginal Community Reference Group (CRG). During the review process, members of the CRG also decided to change its name from ‘Indigenous Reference Group’ to the Aboriginal and Torres Strait Islander Community Reference Group and this change has been reflected throughout the paper.

Page 4 – lines 1-8 – do colonisation and structural racism also create barriers for First Nations people engaging or receiving healthcare..? might be useful to make clear here and cite as well – and this also feeds into the importance of your thoughtful study design including ethical and cultural considerations.

Thank you for the opportunity to add this important detail. We have added to the opening paragraph on page 4:

Aboriginal peoples continue to experience complex health needs and barriers to appropriate health care due to the enduring legacy of colonisation and associated race-based government policies in Australia.[8] These complex health and social needs of are well known and well documented; improving access to high quality health care that meets the needs, values and preferences of Aboriginal peoples is paramount to improving wellbeing.

Needing to have a clinical diagnosis may be challenging with many of the general population underrepresented and Indigenous populations in particular whose frameworks for health, wellbeing and ageing may differ from Western medicalised models (see examples from NZ). Although it appears strong networks are already formed – perhaps some indicators of approximate numbers of First Nations people with dementia in these (or such) clinicals could be useful – to give an idea of the pool of potential participants..?

We are cognizant of the fact that dementia is poorly diagnosed and documented among Aboriginal and Torres Strait Islander people, especially in Aboriginal-specific primary care. Although we do have some data collected from audits conducted at 12 Aboriginal Community Controlled Health organisations this data is not currently public/published and as such we are unable to provide this level of data into our protocol.

We are also unsure as to which organisations and health services providers will be included in this study and therefore, cannot accurately estimate the total pool of potential participants.

Page 11 – line 35 – can they still take part if they want to participate but not have audio recording..? – e.g. could the researchers note-take and still include their yarns?
Perhaps there is space to consider identifying any potential limitations and how the study has been designed to mitigate these

We will not exclude participants should they not wish to have their yarn audio recorded. We have added some detail at the end of the data collection section.

Research yarns will be audio-recorded with participants' consent and those who do not wish to be audio-recorded will not be excluded from participating. Researchers will take detailed notes should participants not consent to their yarn being audio-recorded.

Overall, an exciting project and well written comprehensive protocol. If space (and appropriate) could be nice to include an example of the kind visual output described on page 10

Visual outputs will be guided by each participants preferences, hence we are not able to provide an example here. We will be sure to provide many visuals in our results papers.

Reviewer 2

Introduction

Question: Page 4 line 42 – 44 – these modifiable risk factors and comorbid conditions are also closely linked to the ongoing impacts of colonisation, racism and discrimination/inequities. A sentence could be added referring to this, to enable the authors/project to actively counter the blaming of First Nations people for the position they find themselves in. I note you have discussed issues of access to care.

We have addressed this comment in our response to reviewer 1's feedback. Please see additional comment on the impact of colonisation in the opening paragraph on page 4.

Methods

Question:Page 7 Figure 1. Will/have the IRG also have/had input into the design of the research, & or PJM process/ tools as per statement bottom of page 12.? Perhaps another circle of IRG input could be placed before Phase 1 and after Phase 3 – this could be with a dashed circle to show overall governance of the project and processes and well as intensive involvement in the PJM specifically?

The OnTRACK Community Reference Group (IRG) has already had input into the design of this research, therefore we agree with the suggestion to add additional circles to the figure. We have amended the figure to reflect these suggestions.

Well described extension of the research yarn that is very responsive to each person/family/community. I can't wait to read the articles resulting from this work!

Thank you 😊

Question: Date range – I may have missed this, but I cannot locate the dates that this project will be undertaken.

Thank you for the opportunity to add this important detail. We have added this into the first line of the methods section.

Question: Cultural background/ experience/ roles of research team and authors. These details may have been included in other submitted documentation, and/but an included positioning of all authors, including cultural background will strengthen the paper and inform readers as to the level of First Nations researcher/community member/ lived experience and involvement.

The authors agree that it is important to include this information regarding researcher roles and backgrounds, especially in the context of Aboriginal health research and qualitative research methods. We have added some detail around the authorship team's cultural backgrounds.

Our authorship team includes two Aboriginal researchers (DB, a Bard/Yjindjarbandi woman and senior research leader; IK, a Warrawong doctor)

Considering we are still in the planning phases of this research, we are unsure exactly who will be involved and in what roles. We will provide an in-depth positionality statement within any papers that result from this work.

Reviewer: 3

Thank you for the opportunity to review this well-written protocol paper - it clearly articulates the project in a culturally secure manner and provides excellent detail of the nuances of working with First Nations people. A couple of minor points to address:

Pg 9, line 26 - how will diagnosis be confirmed?

Thank you for the opportunity to add this detail. Diagnosis will be confirmed by the local treating team and/or medical records.

Participants in this group will have a diagnosis of cognitive impairment or dementia (as diagnoses by their treating clinician or/and medical records), therefore advice from the local treating team/a trained clinician will be sought to determine if potential participants are able to be approached (i.e. permission to access the person with dementia) by the research team.

Pg 13, lines 38-40, the emphasis on resources is on ACP/EOL - the aims of the project are to inform high-quality care overall, so is there any consideration of dissemination for this? Perhaps it is embedded within the remaining section of dissemination, but the opening sentence focuses only on ACP/EOL

We have amened this section to better reflect our aims.

Findings from this study will inform the development of comprehensive best practice guidelines on culturally responsive dementia care including challenges relating to multimorbidity and approaches to ACP and end of life care.

There are a few small grammatical corrections to be made - pg 3 line 41, pg 4 line 14 and 36, line 53 in box 1.

We have addressed grammatical errors throughout the manuscript.
